# Income mortality paradox by immigrants’ duration of residence in Sweden: a population register-based study

**DOI:** 10.1136/jech-2023-220500

**Published:** 2023-09-05

**Authors:** Alexander Miething, Sol P Juárez

**Affiliations:** Department of Public Health Sciences, Stockholm University, Stockholm, Sweden

**Keywords:** MORTALITY, INEQUALITIES, HUMAN MIGRATION, ETHNIC GROUPS

## Abstract

**Background:**

Studies have shown that, compared with the general native population, immigrants display weaker or absent income gradients in mortality. The aim of this study is to examine the extent to which the income gradient is modified by immigrants’ duration of residence in Sweden.

**Methods:**

Swedish register data from 2004 to 2016 were used to study the association between individual income and all-cause mortality among foreign-born and Swedish-born individuals at ages 25–64 years. Based on relative indices of inequality (RIIs) and slope indices of inequality (SIIs) derived from Poisson regressions, we measured relative and absolute mortality differentials between the least and most advantaged income ranks. The analyses were stratified by sex, immigrants’ European or non-European origin, and immigrants’ duration of residence in Sweden.

**Results:**

The relative income inequality in mortality among immigrant men was less than half (RII: 2.32; 95% CI: 2.15 to 2.50) than that of Swedish-born men (RII: 6.25; 95% CI: 6.06 to 6.44). The corresponding RII among immigrant women was 1.23 (95% CI: 1.13 to 1.34) compared with an RII of 2.75 (95% CI: 2.65 to 2.86) among Swedish-born women. Inequalities in mortality were lowest among immigrants who resided for less than 10 years in Sweden, and most pronounced among immigrants who resided for more than 20 years in the country. Corresponding analyses of absolute income inequalities in mortality based on the SII were largely consistent with the observed relative inequalities in mortality.

**Conclusions:**

Income inequalities in mortality among immigrants differ by duration of residence in Sweden, suggesting that health inequalities develop in the receiving context.

**WHAT IS ALREADY KNOWN ON THIS TOPIC**

- Immigrants often exhibit a mortality advantage relative to the native population that tends to disappear with their time spent in the country.
- Evidence also suggests that immigrants show smaller income inequalities in mortality compared with the native-born population (the so-called ‘income mortality paradox’).

**WHAT THIS STUDY ADDS**

- Compared with native Swedes, immigrants in Sweden show a mortality advantage, and also experience an income mortality paradox upon arrival that is modified by duration of residence.
- Our findings show that immigrants’ mortality advantage and their income inequalities in mortality differ by duration of residence, suggesting a possible converge with the levels of the native-born population.

**HOW THIS STUDY MIGHT AFFECT RESEARCH, PRACTICE OR POLICY**

- Future research should identify the mechanisms through which (income) inequalities in health and mortality among immigrants develop in the receiving country.

## Introduction

Immigrants in high-income countries, including Sweden, have consistently exhibited a migrant mortality advantage relative to the native-born majority population, despite experiencing socioeconomic disadvantages upon arrival.^1 2^ Yet, this is not the only puzzling observation regarding migrants’ mortality that challenges our understanding of health inequalities in this group. Studies have shown that, compared with the general native population, immigrants display weaker or absent income gradients in mortality.^3–5^

The most prevailing explanation for the weak income gradient in mortality among immigrants is connected to the process of self-selection that characterises the migration phenomenon.^6 7^ It is postulated that persons who migrate are better equipped than ‘stayers’ with resources and health conditions necessary for the challenging transfer between countries. Thus, such health protections conferred in origin, combined with advantages acquired in the receiving country (like increases in absolute income, welfare benefits and access to healthcare), may counteract the adverse health effects of income disadvantages experienced upon arrival, leading to a mismatch between income and health in this group.

Immigrants’ relative health advantage has been shown to attenuate with increasing time in the receiving context,^8 9^ while at the same time their socioeconomic conditions on average tend to improve with longer duration of residence.^10 11^ The extent to which both phenomena contribute to (ie, amplify or mitigate) income-related inequalities in mortality has not been examined in previous research. Moreover, despite increased absolute income over time, growing awareness of relative social inequalities in the destination context^12^ may exacerbate the income gradient in health and mortality among this group.

Relative inequalities emphasise the relational nature of disadvantages by situating individuals’ income position in relation to the population at large, and presumably capture the psychosocial mechanisms through which inequalities are embodied.^13 14^ Although studies on relative inequalities in health among immigrants are scarce, there is evidence supporting the role of psychosocial factors, for example, by showing the adverse health consequences of a mismatch between expectations and experiences. Immigrants are more likely than natives to suffer from socioeconomic status discrepancies (ie, mismatches between education, occupation and income) that have been shown to translate into worse health.^15 16^ Increasing relative inequalities among immigrants can translate into further health and social inequalities among their descendants and in the society at large.

With consideration to the aforementioned phenomena, the aim of this study was to examine relative and absolute income inequalities in mortality by immigrants’ duration of residence in Sweden, using the native (Swedish-born) population as the reference group.

## Data and methods

This study uses data from longitudinal national registers that comprise the total population registered in Sweden from 2004 to 2016. Pseudonymised unique personal identifiers allowed us to link mortality records from the total population and cause of death registers to the Longitudinal Integration Database that contains sociodemographic data and information on individuals’ region or country of origin.

### Study sample

The study included foreign-born and Swedish-born individuals aged 25–64 years at any time during the study period. This population was followed until 2016 using an open cohort design, which facilitated the inclusion of new individuals who immigrated or reached 25 years of age during the study. We right-censored individuals who were above age 64 years or emigrated during the study period. Individuals with missing information on income (n=168 843) were excluded from the analysis. The final study sample comprised 6 450 708 individuals.

### Study variables

We distinguished between foreign-born individuals (broadly categorised as ‘immigrants’) and the native-born population in Sweden. Immigrants were further distinguished by European and non-European origin and their duration of residence in Sweden, that is, whether they resided for less than 10, 10–20 or more than 20 years in Sweden.

### Exposure variable

Individuals’ disposable net income served as the exposure in this study. We used a 3-year average of individuals’ latest recorded annual income to reduce possible biases induced by use of income from a single year. Income information in the registers was collected in November of each year. Accordingly, the income information refers to the calendar years prior to individuals’ year of death, unless the death occurred in December. The timing of the income assessment was consistent for both immigrants and native Swedes, ensuring comparability.

### Outcome and covariates

The main outcome was all-cause mortality, which serves as a general measure of health at the population level and was assessed from 1 January 2004 to 31 December 2016. We also calculated age-specific mortality rates (ASMRs) per 100 000 person-years using the Swedish population from 2016 as the standard population. The covariates included individuals’ age, distinguishing between four age groups (25–34, 35–44, 45–54 and 55–64 years of age), and their highest achieved educational level, categorised into primary, secondary and tertiary degrees.

### Statistical analysis

The study investigated income-related mortality among foreign-born individuals and the native-born population in Sweden. Longitudinal data were used to assess the cross-sectional association between income and mortality at the most recent observed time point, either when individual observations ended or when individuals died. A rank-based approach was chosen to estimate the ‘relative index of inequality’ (RII) and the ‘slope index of inequality’ (SII) to measure relative and absolute income inequalities in all-cause mortality in population subgroups, respectively. RII and SII are established instruments to study socioeconomic inequalities in relation to health outcomes.^17 18^ They account for the entire distribution of continuous variables, are not affected by sample sizes and thus suitable for comparing health inequalities between social groups.^19^ The RII has a linearity assumption that might be violated in some of the studied subgroups as the graphical inspection of the plotted associations between income ranks and mortality indicates (online supplemental figures 1 and 2). This issue is further discussed in the Strengths and limitations section.

10.1136/jech-2023-220500.supp1 Supplementary data

The calculation of the RII and SII involves several steps: first, we ranked individuals by the average of their latest incomes within the overall income distribution. The ranking was performed separately by sex, age group (using 5-year age bands), and calendar year to account for the varying incomes in these groups and over time. Subsequently, we standardised the obtained rank values by dividing them by the number of individuals in each age-specific, sex-specific and calendar year-specific subgroup. This procedure ensured that income ranks were not distorted by different sizes or distributions of age and sex in the studied subgroups. The resulting ranks are equivalent with cumulative rank probabilities, also known as ‘ridits’, which accounted for the proportional distribution of the population in each subgroup. These rank probabilities (ridits) ranged from ‘0’ for the least deprived to ‘1’ for the most deprived income rank.

Next, the ridit scores were employed as predictor variables in Poisson regression models. The obtained incidence rate ratios are equivalent with the RII that denotes the relative mortality risk for the lowest income rank compared with the highest income rank within the age-specific and sex-specific income distribution among the foreign-born and native-born population. RIIs larger than 1 indicate a negative relationship between income and mortality, indicating income deprivation. The higher the RII exceeds 1, the greater the degree of income inequality in relation to mortality. Conversely, RIIs below 1 indicate a positive relationship between income and mortality, with lower income predicting a lower risk of mortality.

In a third step, we calculated SIIs that accounted for absolute income inequalities in mortality on the population level. The SIIs were derived from RIIs and ASMRs and denoted the absolute differences in ASMRs per 100 000 person-years between the most and least advantaged income rank positions. The formula 2×ASMR×(RII−1)/(RII+1) was used to calculate the SII.^20^

All analyses were performed with Stata V.15.1. Python V.3.10 and the Matplotlib library V.3.5.2 were used to create figures.

### Patient and public involvement

Neither patients nor the public were involved in this study.

## Results

Table 1 presents the distribution of study variables, including group-specific numbers of deaths and income levels. The figures demonstrate that immigrants generally have lower incomes, lower mean age and more pronounced educational differences (holding more primary and tertiary degrees) compared with native Swedes. The majority of immigrants have resided in Sweden for more than 20 years. The proportion of non-European immigrants is slightly higher than that of individuals with European descent. Additional information about income ranges can be found in the online supplemental table 3.

**Table 1:** Descriptive characteristics of the study population, 2004–2016

	Foreign-born				Native-born (Swedish)			
	%	No of deaths	Income rank probability*	Absolute income (mean in 1000 Swedish kronor)	%	No of deaths	Income rank probability*	Absolute income (mean in 1000 Swedish kronor)
All individuals	n=1 360 863	19 276	0.66	158.7	n=5 089 845	112 040	0.47	244.4
Sex								
Men	48.5	11 854	0.66	171.3	51.1	68 565	0.47	271.8
Women	51.5	7422	0.65	146.3	48.9	43 475	0.47	215.7
Age group								
25–34	28.3	1089	0.69	121.1	23.6	5985	0.45	205.1
35–44	28.3	1987	0.65	146.3	25.5	10 118	0.46	223.8
45–54	24.8	5134	0.66	176.4	25.3	25 794	0.47	262.6
55–64	18.7	11 066	0.63	196.8	25.6	70 143	0.49	262.9
Educational level								
Primary	22.0	6316	0.74	128.1	13.2	33 668	0.58	204.6
Secondary	43.6	9220	0.65	161.3	55.3	60 523	0.49	235.6
Tertiary	34.4	3740	0.61	173.4	31.6	17 849	0.39	276.5
Duration of residence								
Less than 10 years	32.8	2062	0.75	109.0				
10–20 years	24.7	3335	0.66	152.9				
More than 20 years	42.5	13 879	0.57	204.2				
Region of origin								
European	47.5	13 278	0.60	183.1				
Non-European	52.5	5998	0.70	138.2				

*The rank probabilities (‘ridits’) ranged from ‘0’ for the least deprived to ‘1’ for the most deprived income rank. Therefore, higher rank probabilities correspond to lower (absolute) income and vice versa.

**Figure 1:**
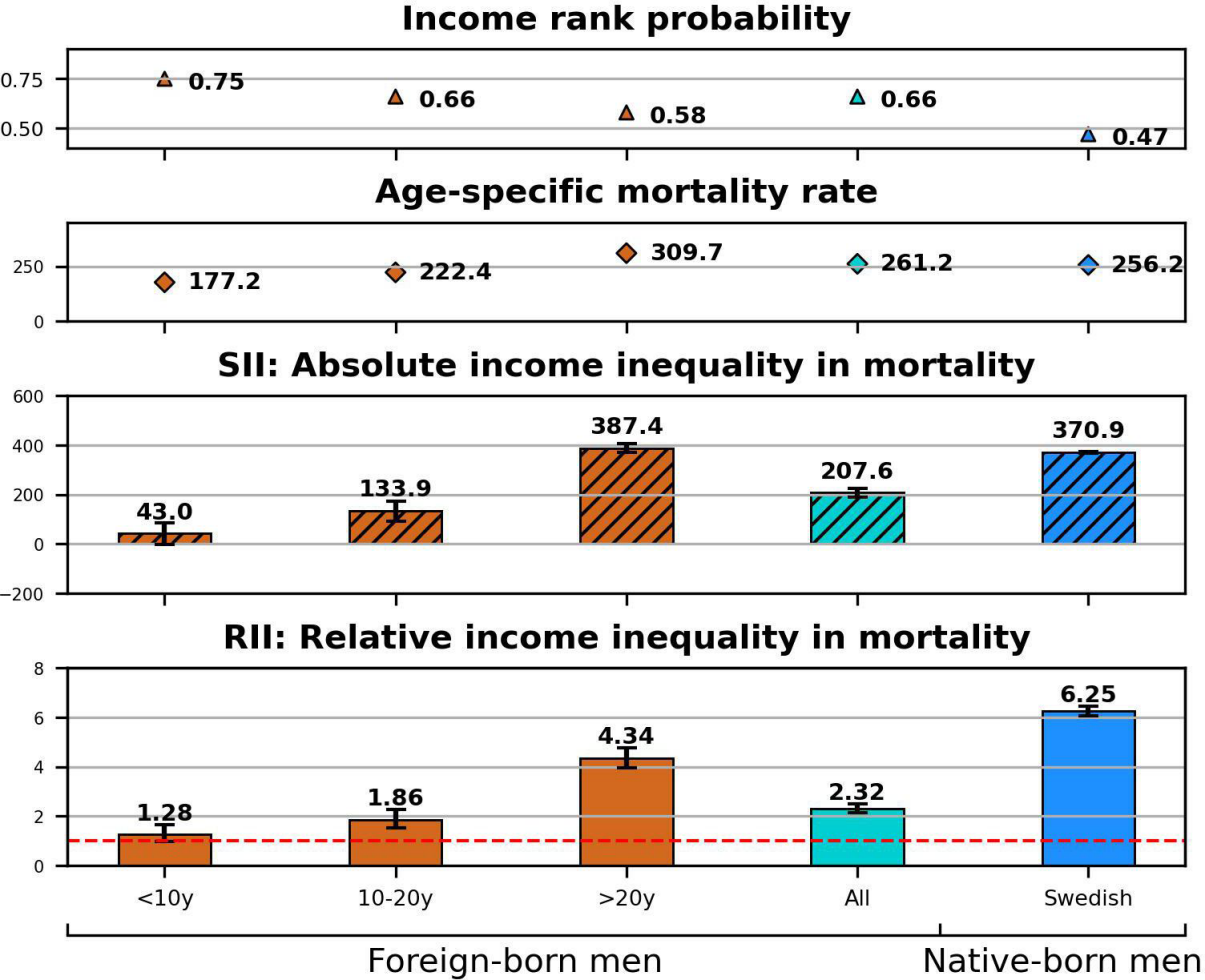
Men’s relative income rank probabilities, age-specific mortality rates, absolute and relative income inequalities in mortality by nativity background and duration of residence. RII=1 indicates the absence of income inequalities in mortality (dashed red line). RII, relative index of inequality; SII, slope index of inequality.

**Figure 2:**
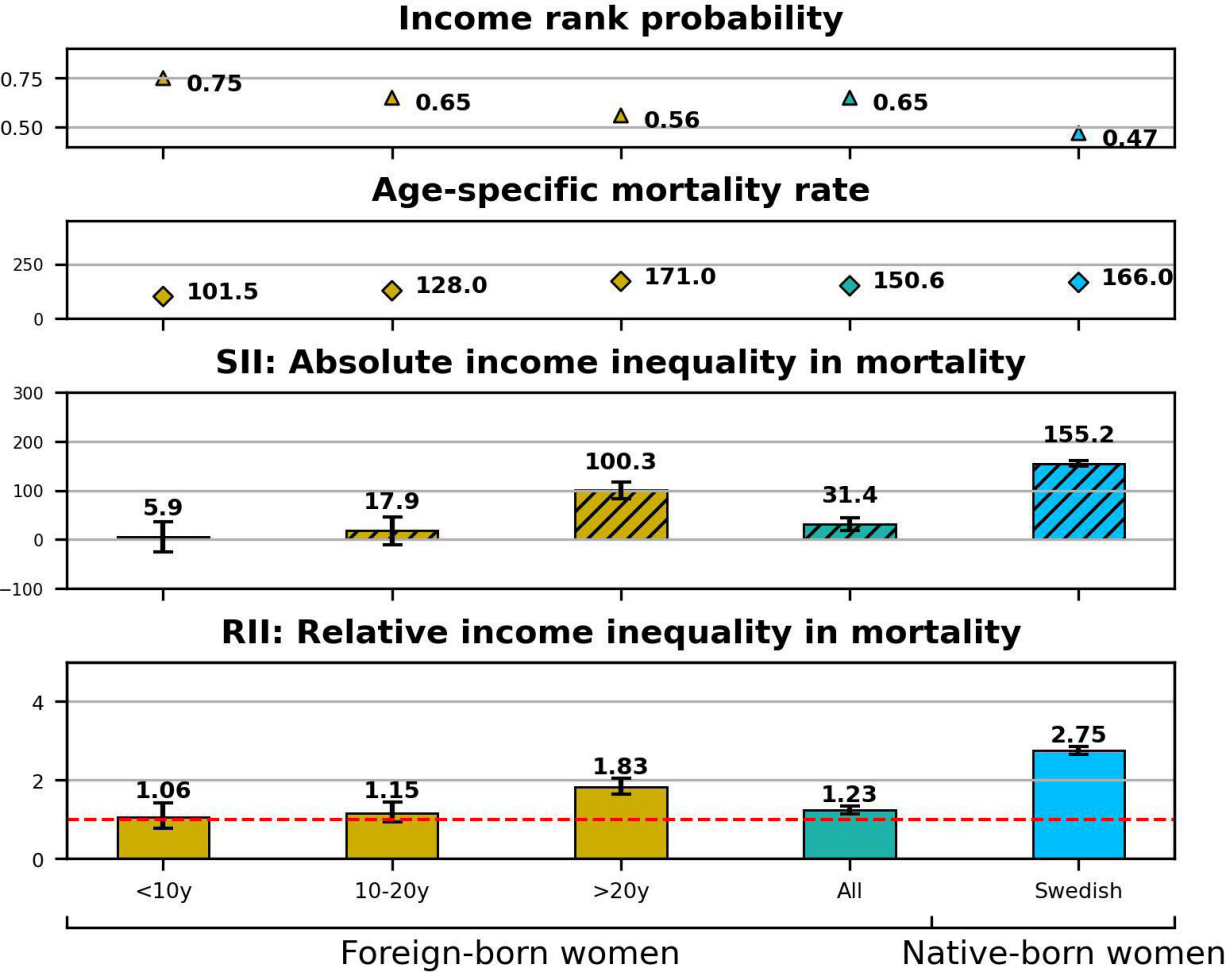
Women’s relative income rank probabilities, age-specific mortality rates, absolute and relative income inequalities in mortality by nativity background and duration of residence. RII=1 indicates the absence of income inequalities in mortality (dashed red line). RII, relative index of inequality; SII, slope index of inequality.

**Figure 3:**
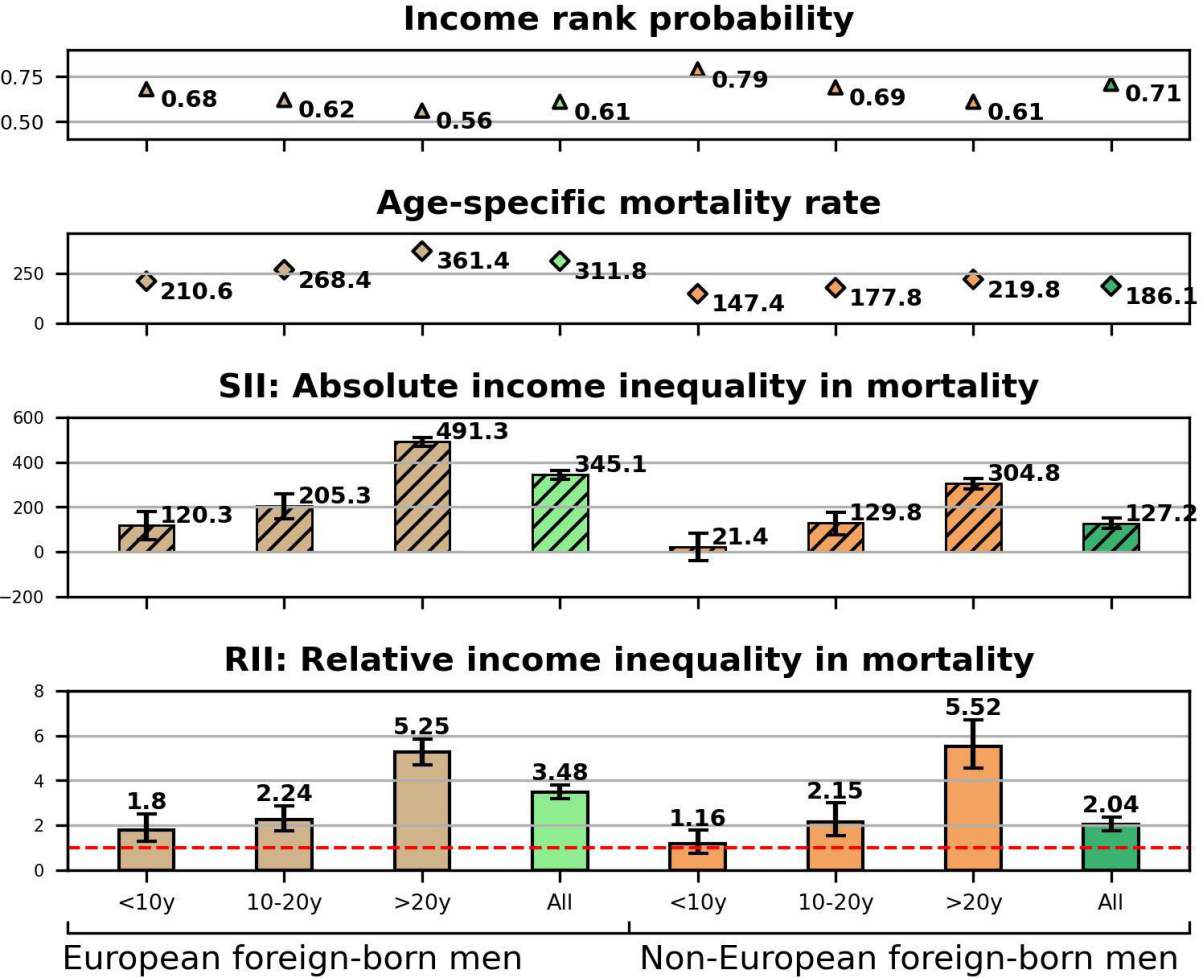
Men’s relative income rank probabilities, age-specific mortality rates, absolute and relative income inequalities in mortality by region of origin and duration of residence. RII=1 indicates the absence of income inequalities in mortality (dashed red line). RII, relative index of inequality; SII, slope index of inequality.

**Figure 4:**
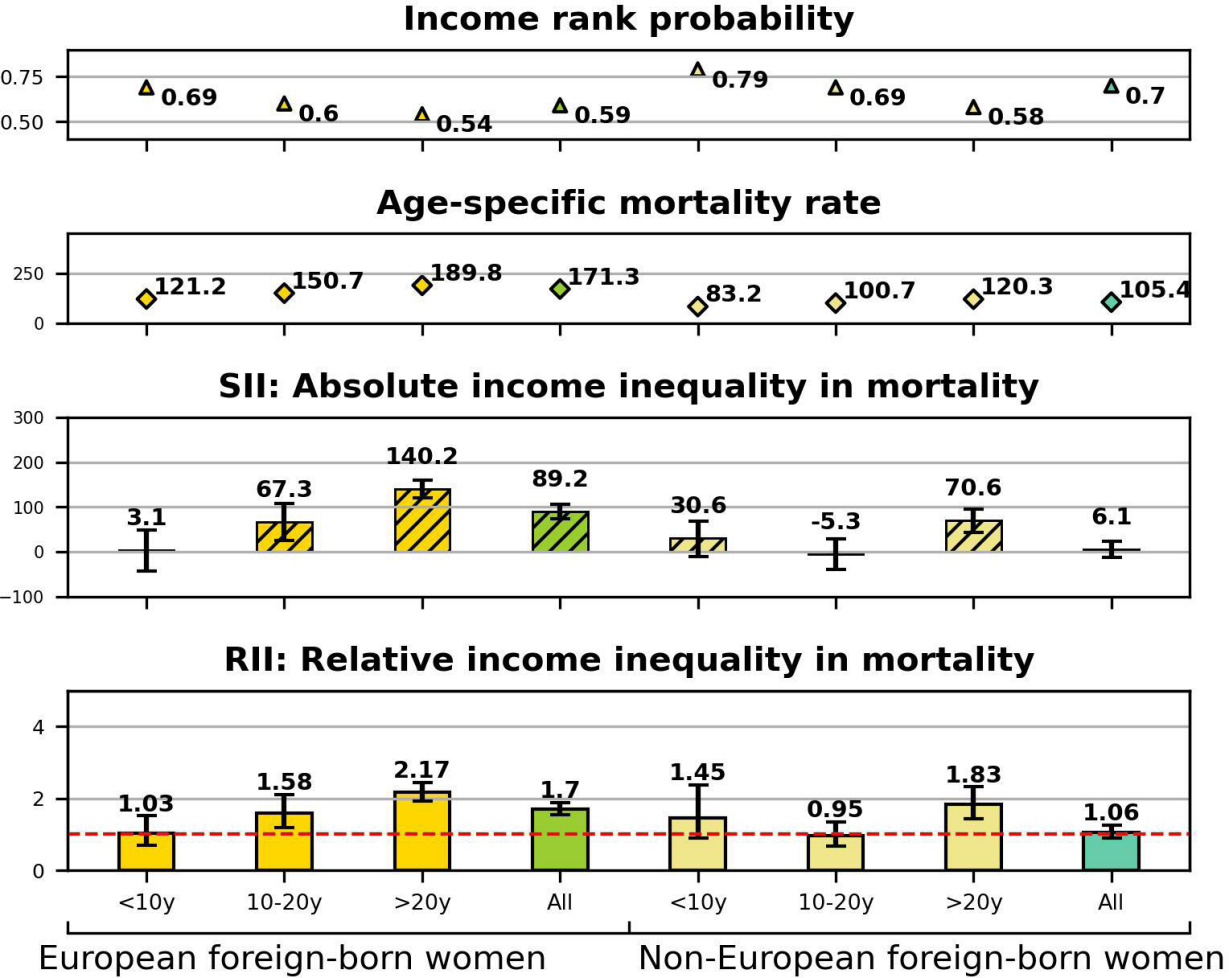
Women’s relative income rank probabilities, age-specific mortality rates, absolute and relative income inequalities in mortality by region of origin and duration of residence. RII=1 indicates the absence of income inequalities in mortality (dashed red line). RII, relative index of inequality; SII, slope index of inequality.

The results from age-adjusted and education-adjusted Poisson regressions as well as information on individuals’ cumulative income rank probabilities and age-specific mortality rates are presented in the figures 1–4. Point estimates with CIs before and after adjustment for education are provided in the online supplemental tables 4 and 5. The bar charts at the bottom of the figures display SIIs and RIIs showing the absolute and relative level of income-related mortality in each population subgroup differentiated by nativity, duration of residence, and European or non-European origin.

Both foreign-born men (figure 1) and women (figure 2) demonstrated less advantaged income rank probabilities (ridits men: 0.66; women: 0.65) compared with native Swedes (ridits men: 0.47; women: 0.47). The figures also show a downward trend in income rank probabilities (as per ridits) by immigrants’ duration of residence in Sweden.

Immigrant men (figure 1) showed slightly elevated ASMR (261.2) compared with Swedish-born men (ASMR: 256.2). However, immigrant men who resided less than 10 years in Sweden demonstrated comparably low mortality rates (ASMR: 177.2); among women, this mortality advantage was more pronounced (figure 2): female immigrants on average showed lower mortality rates (ASMR: 150.6) compared with Swedish-born women (166.0).

The highest degrees of relative and absolute income inequality in mortality were detected among the Swedish-born (figures 1 and 2). Immigrants showed on average significantly lower RIIs (2.32; 95% CI: 2.15 to 2.50) and SIIs (207.6; 95% CI: 190.5 to 224.1) but with a clear ascending trend by duration of residence. Women (figure 2) showed a markedly lower magnitude of income-related mortality risk compared with men (figure 1), but nevertheless a similar pattern emerged between the foreign-born (RII: 1.23; 95% CI: 1.13 to 1.34) and Swedish-born (RII: 2.75; 95% CI: 2.65 to 2.86) women.

Figures 3 and 4 display findings for immigrants distinguished by European and non-European origin. European immigrant men (figure 3) on average showed more advantaged income rank probabilities (ridits: 0.61) compared with their non-European counterparts (ridits: 0.71) and retained this advantage during the post-migration period.

Increasing mortality rates by duration of residence (ASMR) in figure 3 confirm the decline in the mortality advantage of immigrants shown in figure 1. While the relative inequalities in mortality (RII) by duration of residence were similar between those of European and non-European origin, absolute inequalities were more pronounced among the former.

In figure 4, we see larger inequalities among women with European origin (RII: 1.70; 95% CI: 1.54 to 1.89) compared with nearly absent inequalities among non-European women (RII: 1.06; 95% CI: 0.89 to 1.26). Similar to men, European women’s income-related mortality increased by duration of residence. Non-European women in contrast did not show the marked increase in income-related mortality by duration of residence as seen in the other subgroups.

### Sensitivity analysis

Sensitivity analyses were conducted to demonstrate that differences in income-related mortality between native Swedes and immigrants remained unaffected by variations in age compositions (online supplemental table 6). Furthermore, the table reveals that income-related mortality among Swedes did not substantially change even when second-generation immigrants (n=523 731) were excluded from the analysis.

## Discussion

This study examined the income mortality gradient among immigrants in Sweden when using the Swedish-born native population as a comparison group. Our findings show that the income mortality gradient among immigrants was larger among those with longer duration of residence, although without reaching the levels of the native population. Stratified analysis revealed a significant variation by immigrants’ origin (European or non-European) and gender. Income-related mortality was more pronounced among immigrants with European origin and also differed by immigrants’ duration of residence. Long-term immigrants who resided more than 20 years in Sweden demonstrated notably higher magnitudes in income-related mortality as compared with more recent immigrants. These patterns were consistent in both men and women, although the income–mortality relationship was less pronounced among women.

### Originality

To the best of our knowledge, this is the first study to examine the income mortality paradox among immigrants in relation to their duration of residence in Sweden. We found that relative inequalities in mortality developed in the destination context for men and women from European and non-European origins.

### Relation to previous studies

Our findings are consistent with previous studies that report a flattened income gradient in mortality among immigrants.^3–5^ Previous studies have also shown that the immigrant health advantage tends to disappear with increasing duration of residence in the receiving society.^8 9^ In line with these findings, the current study suggests a possible increase in immigrants’ mortality by duration of residence. Our findings contribute knowledge by showing that health inequalities also develop with longer time spent in Sweden. This evidence is particularly relevant when discussing the mechanisms behind immigrants’ health deterioration. Specifically, studies have concentrated on the role of the adoption of health risk behaviours under the umbrella of the ‘acculturation paradox’ that emphasises immigrants’ adoption of norms and values of the general population.^21^ Our study emphasises the role of socioeconomic inequalities in the adoption of health risk behaviours, in line with the idea of an ‘unequal assimilation’,^22^ which argues that the integration process is socioeconomically segmented, meaning that immigrants tend to adopt the habits and behaviours that are more common among the low socioeconomic native group.

### Implications for future research

The mismatch observed between higher averaged income and increasing *income inequalities in mortality* by duration of residence is a novel finding that deserves further attention. Upward trends in income may reflect improvements in labour market attachment (ie, participation in the labour market), while income inequalities in mortality can reflect relative disadvantages in job security and expectations.^23^ For example, refugee immigrants in Sweden (included in the non-European category) might experience a progressive independence from state support with increasing duration of residence but such independence could come with the cost of job precaritisation with adverse health effects.^24 25^ For their part, labour immigrants (mostly Europeans in this study) might experience a glass ceiling in the labour market compared with their Swedish native counterparts, leading to increasing inequalities in mortality.^26^

The fact that income improves by duration of residence, while income inequalities in mortality increase, may suggest that health plays a relevant and independent role in conceptualising inequalities and, consequently, should not be viewed solely as a result of social inequalities. Health in general and mortality specifically can capture other forms of inequality that are not necessarily expressed in income differences but rather in other forms of social stratification.^11 27^ This reasoning supports sociological claims that socioeconomic status information (mainly by not exclusively occupation, income, education) should not be considered interchangeably.^28^

The observed differences in income-related mortality between European and non-European immigrants suggest that socioeconomic and sociocultural proximity between country of origin and country of destination influences the nature of the income–mortality relationship. As pointed out in Read *et al*,^29^ the effect of self-selection is more pronounced for (non-European) immigrants from culturally and geographically distant countries, as their migration costs are higher. Non-European immigrants, in particular those from low-income contexts, can also be expected to gain health benefits from an improvement of their absolute material circumstances in the destination context despite occupying relatively low-income ranks. A possible reason is that refugees—usually originating from outside Europe—often receive welfare state provisions. European immigrants, in contrast, tend to originate from contexts that are more similar to Sweden and may be better equipped with the necessary qualifications required for smoother labour market integration. This involves a more seamless socioeconomic transition compared with non-European immigrants but also entails a similar income-related mortality risk as that of the Swedish majority population.

Future studies should evaluate the extent to which the children of immigrants (second generation) experience a full convergence with the levels of health inequalities observed in individuals of Swedish parentage. This examination is essential to evaluate how health inequalities develop over generations.

### Strengths and limitations

A major strength of this study was the use of longitudinal register data with a high coverage of the residential population in Sweden. Another advantage of the register-based study material was the low degree of missing income data and the absence of selection effects due to attrition and reporting biases.

Nevertheless, the study was also subject to limitations. Given that healthy immigrants are more likely to emigrate and return migration is more prominent with increasing duration of residence,^30^ health selection might have affected our results to some extent. Additionally, despite the longitudinal set-up of the study, the association between income and mortality was assessed cross-sectionally. As a result, we cannot determine the direction of the relationship between income and mortality, making it challenging to draw conclusions about the extent of the causal effects of income on mortality. Another aspect of health selection involves the decline of health before death, which could contribute to reduced incomes. Although we used a 3-year average of income to mitigate this effect, it is still possible that it increased the shown RIIs. Further, possible lagged effects of income inequality as well as the autocorrelative nature of individuals’ income positions over time may have distorted the shown associations. A further limitation is the violation of the linearity assumption of the RII. However, despite this limitation, the RII remains suitable for capturing and quantifying *overall* group differences in inequalities. Recent empirical demonstrations also indicate that a violation of the normality assumption (non-normality of residuals) rarely poses significant issues for hypothesis testing.^31^ Another limitation relates to the inclusion of the second-generation immigrants into the Swedish native group. Although officially not immigrants, their foreign background might entail that they are subject to specific forms of inequalities that we do not consider in this study.

## Conclusions

The weak income inequalities in mortality observed for the immigrant population are supported by previous studies in the so-called ‘income mortality paradox’. Yet, our study also shows that income inequalities in mortality differ by duration of residence in Sweden, suggesting that health inequalities could possibly develop in the receiving context. Taken together, these results highlight the dynamic nature of inequalities in the receiving context and how health inequalities emerge in the society at large.

## Data Availability

Data may be obtained from a third party and are not publicly available. Data may be obtained from a third party and are not publicly available. The data used in this study were collected from Swedish administrative registers and can be requested for research use from Statistics Sweden and the Swedish National Board of Health and Welfare.
