# Supplementary material for: Income mortality paradox by immigrants’ duration of residence in Sweden: a population register-based study
Source: J Epidemiol Community Health. 2023 Sep 5;78(1):11–7. doi: 10.1136/jech-2023-220500 (PMC10715552; doi:10.1136/jech-2023-220500)
Supplement: Supplementary data [file jech-2023-220500supp001.pdf]

Supplementary Figure S1: Crude mortality rate by quantiles of income rank, men aged 25-64

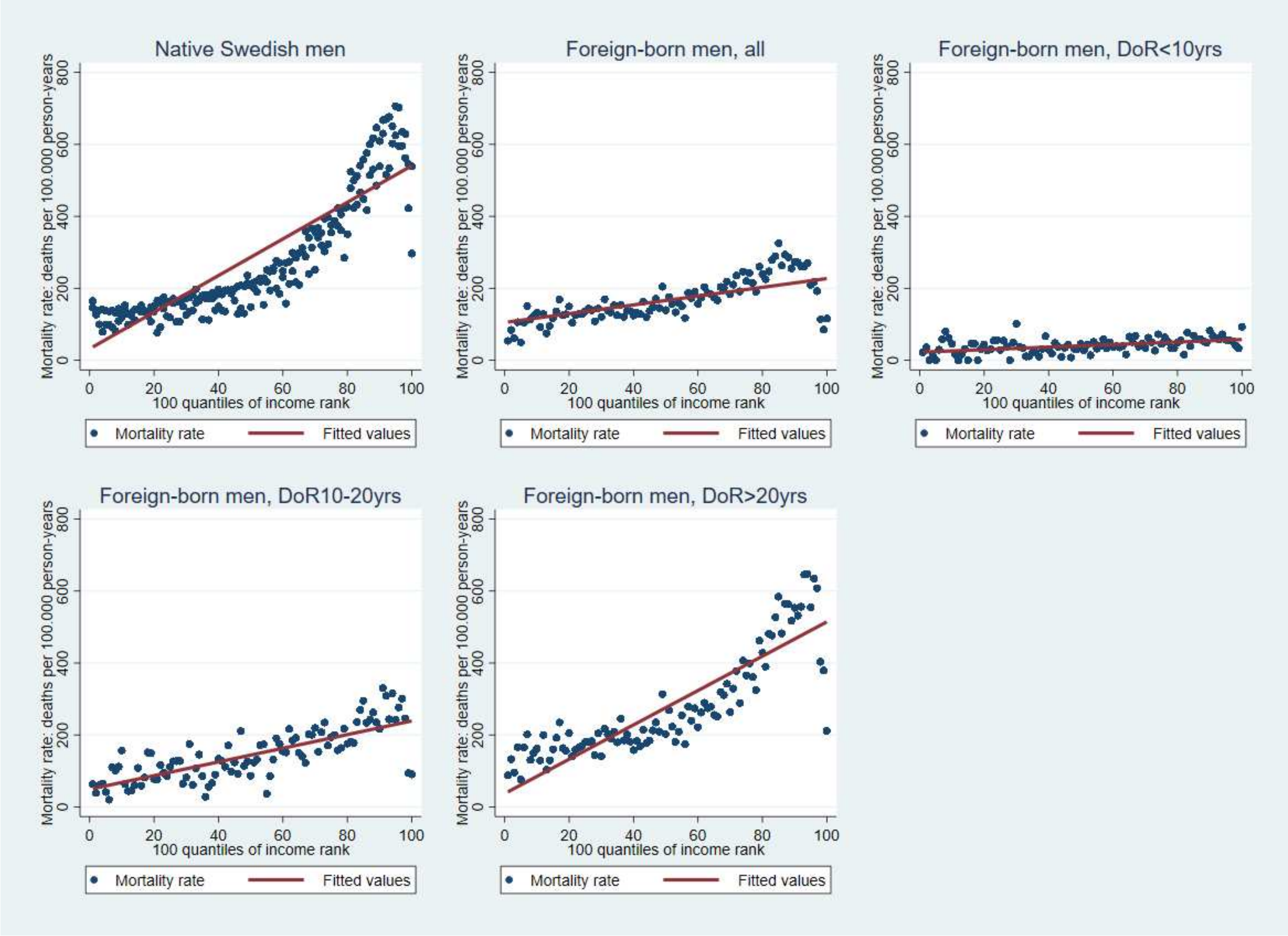

Supplementary Figure S2: Crude mortality rate by quantiles of income rank, women aged 25-64

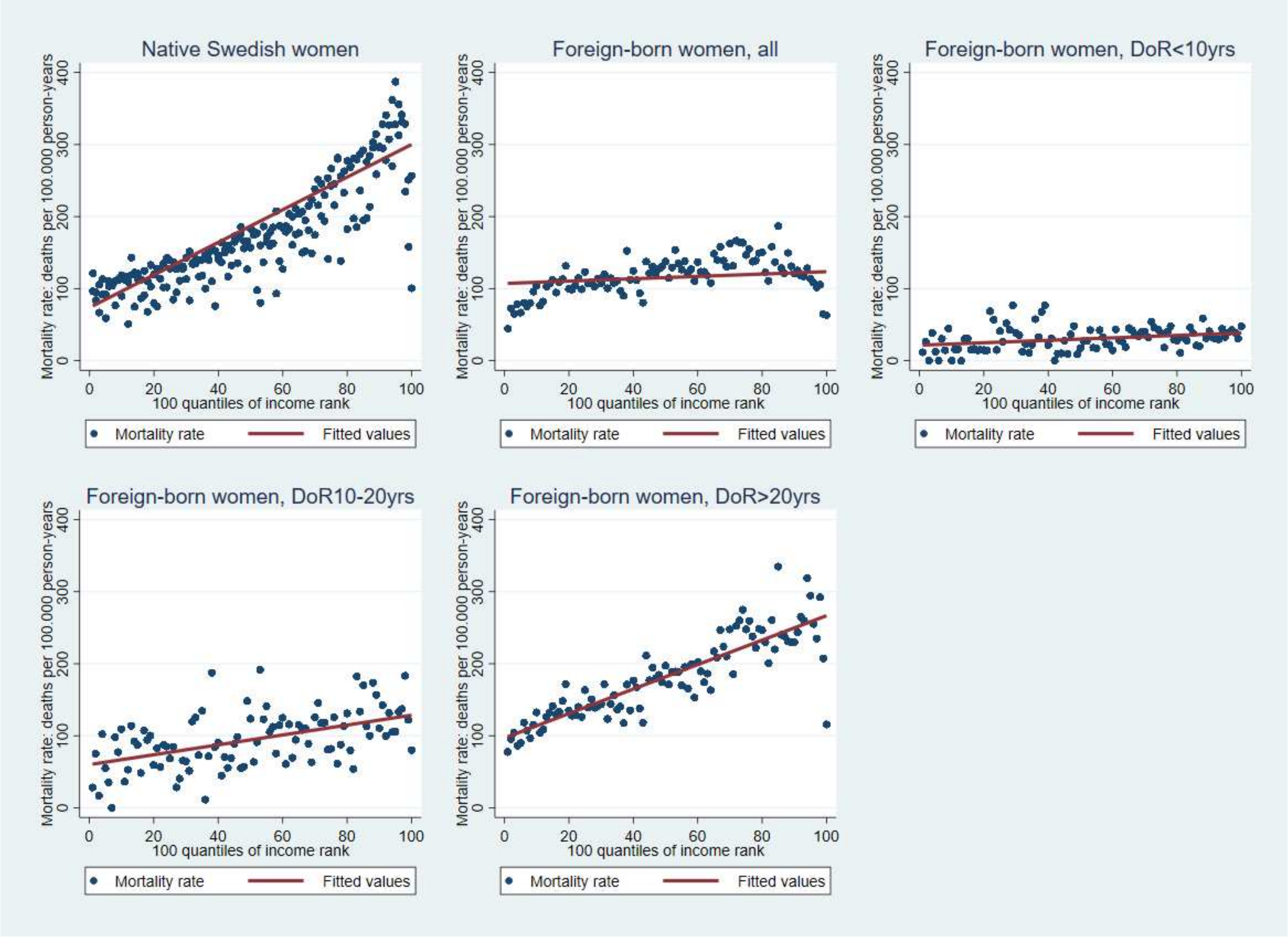

Supplementary Table S3: Additional descriptive characteristics of the study population, 2004–2016

	**Foreign-born**							**Native-born (Swedish)**						
	**%**	**No. of deaths**	**Income rank probability**	**Absolute income** (in 1,000 Swedish kronor)				**%**	**No. of deaths**	**Income rank probability**	**Absolute income** (in 1,000 Swedish kronor)			
				Mean	Std.dev.	1% percentile	99% percentile				Mean	Std.dev.	1% percentile	99% percentile
All individuals	*n=1,360,863*	19,276	0.66	158.7	198.3	0.0	580.4	*n=5,089,845*	112,040	0.47	244.4	376.5	37.2	842.8
Sex														
Men	48.5	11,854	0.66	171.3	233.2	0.0	615.7	51.1	68,565	0.47	271.8	458.3	29.5	938.7
Women	51.5	7,422	0.65	146.3	155.1	0.0	539.4	48.9	43,475	0.47	215.7	261.9	44.3	727.5
Age group														
25-34	28.3	1,089	0.69	121.1	114.1	0.0	434.0	23.6	5,985	0.45	205.1	200.2	19.1	578.5
35-44	28.3	1,987	0.65	146.3	156.1	0.0	546.6	25.5	10,118	0.46	223.8	477.1	28.7	711.3
45-54	24.8	5,134	0.66	176.4	162.9	0.0	639.6	25.3	25,794	0.47	262.6	406.2	43.1	895.5
55-64	18.7	11,066	0.63	196.8	296.3	0.0	681.0	25.6	70,143	0.49	262.9	374.5	50.6	984.4
Educational level														
Primary	22.0	6,316	0.74	128.1	108.6	0.0	404.6	13.2	33,668	0.58	204.6	241.1	24.5	652.9
Secondary	43.6	9,220	0.65	161.3	152.8	0.0	514.1	55.3	60,523	0.49	235.6	356.5	35.9	769.4
Tertiary	34.4	3,740	0.61	173.4	266.8	0.0	687.8	31.6	17,849	0.39	276.5	448.5	48.5	1000.7
Duration of residence														
Less than 10 years	32.8	2,062	0.75	109.0	120.1	0.0	471.9							
10 to 20 years	24.7	3,335	0.66	152.9	142.1	0.0	523.5							
More than 20 years	42.5	13,879	0.57	204.2	254.8	0.0	662.1							
Region of origin														
European	47.5	13,278	0.60	183.1	191.7	0.0	645.5							
Non-European	52.5	5,998	0.70	138.2	201.5	0.0	509.0							

Supplementary Table S4: Relative and absolute income inequalities in mortality 2004-2016: Relative index of inequality (RII; 95% CI) and slope index of inequality (SII per 100,000 person-years; 95% CI) among men aged 25-64.

	**Model 1** (adjusted for age)				**Model 2** (adjusted for age and education)			
	**Relative income inequalities in mortality**		**Absolute income inequalities in mortality**		**Relative income inequalities in mortality**		**Absolute income inequalities in mortality**	
	**RII**	**95% CI**	**SII**	**95% CI**	**RII**	**95% CI**	**SII**	**95% CI**
**Native-born (Swedish) men**	7.60	(7.37-7.82)	393.1	(389.9 - 396.2)	6.25	(6.06 - 6.44)	370.9	(367.2 - 374.6)
**Foreign-born men**	2.62	(2.43 - 2.82)	233.8	(218.1 - 249.1)	2.32	(2.15 - 2.50)	207.6	(190.5 - 224.1)
Duration of residence:								
Less than 10 years	1.40	(1.09 - 1.79)	59.1	(15.7 - 100.8)	1.28	(0.99 - 1.65)	43.0	(-2.2 - 86.7)
10 to 20 years	2.30	(1.90 - 2.79)	175.2	(137.8 - 209.9)	1.86	(1.52 - 2.27)	133.9	(92.4 - 173.0)
More than 20 years	5.17	(4.71 - 5.66)	418.4	(402.5 - 433.4)	4.34	(3.95 - 4.76)	387.4	(369.1 - 404.4)
**European foreign-born men**	3.94	(3.61 - 4.31)	371.2	(353.0 - 388.5)	3.48	(3.18 - 3.81)	345.1	(325.0 - 364.3)
Duration of residence:								
Less than 10 years	2.15	(1.56 - 2.94)	153.4	(92.7 - 207.6)	1.80	(1.30 - 2.50)	120.3	(54.5 - 180.2)
10 to 20 years	2.90	(2.29 - 3.68)	261.6	(210.0 - 307.5)	2.24	(1.74 - 2.87)	205.3	(145.5 - 259.6)
More than 20 years	6.23	(5.61 - 6.93)	522.9	(504.0 - 540.5)	5.25	(4.71 - 5.84)	491.3	(469.6 - 511.5)
**Non-European foreign-born men**	2.27	(1.97 - 2.62)	144.8	(121.9 - 166.5)	2.04	(1.76 - 2.36)	127.2	(102.8 - 150.5)
Duration of residence:								
Less than 10 years	1.23	(0.81 - 1.86)	30.1	(-30.8 - 88.5)	1.16	(0.76 - 1.76)	21.4	(-40.6 - 81.5)
10 to 20 years	2.58	(1.87 - 3.56)	157.0	(107.8 - 199.6)	2.15	(1.55 - 2.99)	129.8	(76.2 - 177.3)
More than 20 years	6.34	(5.24 - 7.67)	319.8	(298.7 - 338.1)	5.52	(4.55 - 6.71)	304.8	(281.1 - 325.5)

Supplementary Table S5: Relative and absolute income inequalities in mortality 2004-2016: Relative index of inequality (RII; 95% CI) and slope index of inequality (SII per 100,000 person-years; 95% CI) among women aged 25-64.

	**Model 1** (adjusted for age)				**Model 2** (adjusted for age and education)			
	**Relative income inequalities in mortality**		**Absolute income inequalities in mortality**		**Relative income inequalities in mortality**		**Absolute income inequalities in mortality**	
	**RII**	**95% CI**	**SII**	**95% CI**	**RII**	**95% CI**	**SII**	**95% CI**
**Native-born (Swedish) women**	3.70	(3.56 - 3.84)	190.7	(186.6 - 194.7)	2.75	(2.65 - 2.86)	155.2	(150.2 - 160.1)
**Foreign-born women**	1.47	(1.35 - 1.59)	57.0	(44.9 - 68.9)	1.23	(1.13 - 1.34)	31.4	(18.5 - 44.1)
Duration of residence:								
Less than 10 years	1.17	(0.88 - 1.57)	16.1	(-13.2 - 44.8)	1.06	(0.78 - 1.43)	5.9	(-24.6 - 35.6)
10 to 20 years	1.47	(1.19 - 1.81)	48.6	(22.0 - 74.1)	1.15	(0.93 - 1.44)	17.9	(-9.9 - 46.2)
More than 20 years	2.24	(2.02 - 2.48)	130.9	(115.7 - 145.5)	1.83	(1.65 - 2.04)	100.3	(83.6 - 116-7)
**European foreign-born women**	2.11	(1.91 - 2.33)	122.3	(107.4 - 136.7)	1.70	(1.54 - 1.89)	89.2	(72.7 - 105.2)
Duration of residence:								
Less than 10 years	1.28	(0.88 - 1.85)	29.5	(-15.2 - 72.3)	1.03	(0.70 - 1.50)	3.1	(-42.9 - 48.8)
10 to 20 years	2.33	(1.77 - 3.06)	120.1	(83.6 - 152.9)	1.58	(1.18 - 2.10)	67.3	(25.0 - 107.0)
More than 20 years	2.70	(2.40 - 3.02)	174.2	(156.4 - 191.0)	2.17	(1.93 - 2.45)	140.2	(120.1 - 159.4)
**Non-European foreign-born women**	1.21	(1.03 - 1.42)	19.9	(2.6 - 36.8)	1.06	(0.89 - 1.26)	6.1	(-11.8 - 23.9)
Duration of residence:								
Less than 10 years	1.54	(0.95 - 2.49)	35.3	(-4.1 - 71.1)	1.45	(0.89 - 2.37)	30.6	(-9.8 - 67.6)
10 to 20 years	1.09	(0.78 - 1.53)	9.1	(-24.7 - 42.4)	0.95	(0.67 - 1.34)	-5.3	(-39.7 - 29.3)
More than 20 years	2.21	(1.76 - 2.78)	90.7	(66.0 - 113.4)	1.83	(1.44 - 2.33)	70.6	(43.6 - 95.9)

BMJ Publishing Group Limited (BMJ) disclaims all liability and responsibility arising from any reliance placed on this supplemental material which has been supplied by the author(s)

Supplementary Table S6: Relative index of inequality (RII; 95% CI) among men and women by age groups, adjusted for age and education

	Men				Women			
	**RII**	**95% CI**	**Mean age**	**Std.Dev.**	**RII**	**95% CI**	**Mean age**	**Std.Dev.**
**Native-born (Swedish)**	6.25	(6.06 - 6.44)	48.8	13.1	2.75	(2.65 - 2.86)	49.1	13.2
Age groups:								
25-34 years	6.18	(5.44 - 7.02)	29.2	2.9	1.75	(1.43 - 2.15)	29.2	2.9
35-44 years	5.09	(4.61 - 5.63)	39.7	2.9	1.45	(1.28 - 1.66)	39.6	2.9
45-54 years	6.89	(6.46 - 7.34)	49.5	2.8	3.24	(3.00 - 3.50)	49.5	2.8
55-64 years	6.20	(5.97 - 6.45)	61.9	2.9	2.95	(2.81 - 3.09)	62.0	2.9
**Native-born without descendants of immigrants (2nd generation)**	6.21	(6.02 - 6.41)	49.5	13.1	2.75	(2.65 - 2.86)	49.7	13.1
**Foreign-born**	2.32	(2.15 - 2.50)	44.2	12.4	1.23	(1.13 - 1.34)	44.7	12.5
Duration of residence:								
Less than 10 years	1.28	(0.99 - 1.65)	37.0	9.2	1.06	(0.78 - 1.43)	36.9	9.3
10 to 20 years	1.86	(1.52 - 2.27)	42.7	10.3	1.15	(0.93 - 1.44)	42.7	10.1
More than 20 years	4.34	(3.95 - 4.76)	51.5	11.7	1.83	(1.65 - 2.04)	51.9	11.5
